# A protocol for a qualitative study on sex trafficking: Exploring knowledge, attitudes, and practices of physicians, nurses, and social workers in Ontario, Canada

**DOI:** 10.1371/journal.pone.0274991

**Published:** 2022-09-27

**Authors:** Danielle Jacobson, Robin Mason, Rhonelle Bruder, Janice Du Mont

**Affiliations:** 1 Women’s College Research Institute, Women’s College Hospital, Toronto, Canada; 2 Dalla Lana School of Public Health, University of Toronto, Toronto, Canada; University of Leeds, UNITED KINGDOM

## Abstract

**Introduction:**

There has been limited research on sex trafficking in Canada from a health and health care perspective, despite U.S. research which points to health care providers as optimally positioned to identify and help those who have been sex trafficked. We aim to better understand health care providers’ knowledge about, attitudes towards, and care of those who have been sex trafficked in Ontario, Canada.

**Methods and analysis:**

Using a semi-structured interview guide, we will interview physicians, nurses, and social workers working in a health care setting in Ontario until data saturation is reached. An intersectional lens will be applied to the study; analysis will follow the six analytic phases outlined by Braun and Clarke. In the development of this study, we consulted the consolidated criteria for reporting qualitative research (COREQ) with regards to reflexivity and study design. We will continue to consult this checklist as the study progresses and in the writing of our analysis and findings.

**Discussion:**

To our knowledge, this will be the first study of its kind in Canada. The results hold the potential to inform the development of standardized training on sex trafficking for health care providers. Results of the study may be useful in addressing sex trafficking in other jurisdictions.

## Introduction

Despite ostensibly being “under-detected”, 32 countries worldwide have reported domestic human trafficking [1, p.11] (that is, human trafficking within a country’s borders) with North American countries reporting increased evidence of those trafficked [2]. While there are multiple types of human trafficking (e.g., forced labour, forced criminal activities, etc.), sex trafficking is a specific form of human trafficking that involves coercion and the “recruiting, transporting or holding [of] victims for sexual exploitation” [3, p.1]. This form of trafficking violates the law, human rights, and also poses health risks such as “physical violence, mental illness”, “violent and unsafe sex practices, inhumane working and living conditions and lack of access to health care services” for those who have been trafficked [4, p.345].

Canada has been recognized as a major trafficking site [5] with Ontario, Quebec, British Columbia, and Alberta reported as “major hubs” for domestic sex trafficking [6, p.2]. Of the 132 sex trafficking charges in Canada between 2007 and 2013 documented by the Royal Canadian Mounted Police [6], Ontario (Canada’s most populous province) had the largest proportion. Differences in provincial reported rates may be related to increased awareness of the issue and not reflect a greater number of cases in this jurisdiction.

While there has been some Canadian research on human trafficking, there has been less on *sex trafficking* specifically [7]. Some studies have focused on counter-human trafficking strategies [8]. Durisin and van der Meulen [9] delved into the shift in discourse (i.e., from a focus on international to domestic human trafficking) within Canada’s legislative efforts to end human trafficking. Others have taken a legal perspective such as Millar and O’Doherty [10]. In the context of predominantly domestic sex trafficking cases, for example, they discussed the ways that anti-trafficking laws overly scrutinize and under protect racial and gender minority groups. Studies of youth who have been sex trafficked have explored the impact on their mental health [11], routes to recruitment and entrapment [12], and higher risk of those within the child welfare system [13]. The limited research on social services for those who have been sex trafficked in Canada has revealed gaps in the delivery of services including a dearth of transitional housing and safe shelters, in addition to barriers to accessing health care, a lack of culturally informed services, and fear of being arrested [14]. Olson-Pitawanakwat and Baskin [15], who examined sex trafficking in the Canadian context, found that Indigenous women rarely accessed mainstream social services and Two-Spirit and trans women felt that such services “rarely understood” them [p. 19].

Canadian research on health and health care for trafficked persons is emergent. A recent review of the scholarly literature on sex trafficking conducted by Hodgins et al. [7], points to survivors’ various mental health challenges (i.e., anxiety, depression) [11], sexual health needs (i.e., care for sexually transmitted infections) [11], and the necessity of emergency medical care [14]. One Ontario study investigated medical students’ awareness of human trafficking (not sex trafficking specifically), finding that students lacked knowledge on the subject [16]. However, research conducted in the U.S. has revealed the many health complications faced by those who have been sex [17, 18] or human [19] trafficked, including physical (e.g., headaches, head injuries, respiratory and gastrointestinal symptoms) [17] and mental health (e.g., post-traumatic stress, trauma, depression, anxiety, flashbacks, low self-esteem) problems [17–19]. When individuals who had been sex and/or labor trafficked sought care, they reported avoiding disclosure of their trafficking status due to shame, anticipated judgmental attitudes of providers, language barriers, and providers’ time constraints [20]. The reluctance to disclose sex trafficking status was also noted by health care providers [21].

Although health care providers are optimally positioned to identify adults who are being trafficked [22], those without training are often less likely to recognize indicators of sex trafficking status [23]. However, little is known about Canadian health care providers’ perceptions of and capacity to respond to adults being sex trafficked. The purpose of this study is to explore the knowledge, attitudes, and practices related to sex trafficking of physicians, nurses, and social workers working in Ontario health care settings. We aim to determine these practitioners’ educational needs and gain a better understanding of factors that influence health care service provision for those who have been sex trafficked. The results hold the potential to inform the development of standardized curricula on sex trafficking for health care providers in Canada and other jurisdictions with the goal of improving services for sex trafficked persons.

## Materials and methods

### Study design

We will employ a qualitative research design [24], and a critical social approach that recognizes that structures and systems influence one’s lived reality, to explore the sex trafficking knowledge, attitudes, and practices of physicians, nurses, and social workers working in health care settings. As individual actors have unique identities as well as experiences, an intersectionality framework [25] will contribute to how race, ethnicity, gender, for example, influence health care providers’ knowledge, attitudes, and care provision practices [26, 27] for patients with varying social identities. The consolidated criteria for reporting qualitative research (COREQ) [28] were used in developing the study (S1 Checklist).

### Recruitment

We will recruit physicians, nurses, and social workers practicing in health care settings in Ontario, Canada’s most populous province. To be included in this study participants will not need to have had experience providing care to a person who has been sex trafficked. Recruitment is expected to begin in the fall 2022 and will continue until data saturation is reached and each of the professional groups has been sufficiently represented [24, 29, 30]. We will utilize convenience sampling by reaching out to our professional networks and to relevant organizations. Snowball sampling–referral to other potential participants by active participants–will also be used. Health care providers will be recruited via relevant listservs and emails to appropriate organizations and networks. We will find relevant organizations through a Google search, targeting the seven regions of Ontario: central, central west, central east, southwest, east, northeast, and northwest. Organization’s general email provided on their web page or a person responsible for research inquiries will be contacted. We aim to include organizations serving urban, rural, and remote populations. Information shared will include study purpose, participation criteria, nature of the interview, and contact information.

### Interviews

One-on-one, in-depth, semi-structured interviews will last approximately one hour. Eligibility to participate will be confirmed prior to the interview. Participants will be sent the Letter of Information, complete a written Informed Consent Form (describing the study purpose, participation and its risks and benefits, and steps taken to maintain confidentiality), and a Sociodemographic Questionnaire (including participant background and details of any past training and education on sex trafficking). These forms will be digitally signed and returned via email. Interviews will take place on zoom and will be audio or video recorded. Participants will be reminded that the interview will be recorded and they can refuse to answer any question or withdraw from the interview at any point without consequences. At the start of each interview, participants will choose a pseudonym which will be used on the transcript files and in any future presentations and publications. Zoom or a similar software will be used to generate transcripts from the interviews. Transcripts will be checked and re-checked against the recording for accuracy by several members of the research team. All information will be de-identified to maintain confidentiality. Each transcript and all associated materials will be maintained in separate digital folders, labeled with the participant’s pseudonym. These materials will be stored on a shared, secure OneDrive folder accessible only by the research team. Recruitment for this study has not yet begun.

### Interview guide

We will ask open-ended questions to elicit information about health care providers’ perceptions of and capacity to respond to adults who have been sex trafficked. Knowledge will be explored through questions such as, “What is sex trafficking?” Attitudes will be explored through questions related to beliefs, including who is likely to be sex trafficked. Practices will be examined in the context of the provision of care and understanding of the providers’ role. Most of the questions in the interview guide were developed to address gaps noted in the literature as well as derived from team members’ expertise. Some items in the interview guide were drawn or adapted from Cunningham and DeMarni Cromer’s [31] Human Trafficking Myths Scale specific to knowledge (e.g., “If someone did not want to be trafficked, he or she would leave the situation”) and attitudes (e.g., “Normal-appearing, well-educated, middle-class people are not trafficked”) about trafficking [31, p.240]. The interview guide will be piloted, reviewed, and any necessary changes for clarity made.

### Data analysis

Interview data will be analyzed according to Braun and Clarke’s [32] six phases of thematic analysis. During phase one, we will repeatedly listen to interview recordings and read and re-read transcripts, writing down preliminary thoughts and ideas. In phase two, initial codes for noteworthy features across all interviews will be generated using a qualitative software package such as Dedoose [33]. During phase three, preliminary codes will be organized into emerging themes. In phase four, we will review, discuss, and refine emerging themes to best reflect patterns in and across the data. During phase five, themes (including content, excerpts and names of themes) will be revised to better reveal the “overall story the analysis tells” [32, p.87]. Finally, in phase six, we will select the richest examples of quotes for each theme and relate the analysis back to the research question and related literature [32]. Saturation will be reached when no new themes emerge. Quality of the study will be evaluated via transferability [34] and resonance [35]. These concepts refer to “a study’s potential to be valuable across a variety of contexts or situations”, considering the cultural influence of the time and place of data collection [35, p.845].

### Researchers’ positionality

Our multidisciplinary research team has extensive expertise in qualitative research and a history of conducting research focused on sensitive topics. We value work that is designed to improve individual and institutional responses to persons who have been sex trafficked. Throughout our study, we will reflect on and challenge one another about the ways in which our professional identities as well as our varied social identities and circumstances [36] may be influencing our research approach, the questions we ask, how we relate to participants, and analytic processes [37].

### Ethics and dissemination

This research was reviewed and approved by Women’s College Hospital Research Ethics Board in December 2021 (REB# 2021-0133-E). In addition to traditional means of dissemination such as publications, presentations, webinars, and infographics, we will also share the findings from this research via social media and on a WebPortal we are currently developing focused on sex trafficking in Canada.

## Discussion

### Contributions

To our knowledge, this is the first study to investigate physicians’, nurses’, and social workers’ perceptions of and capacity to respond to adults who have been sex trafficked in Canada. Applying a critical social approach and intersectional lens to the knowledge, attitudes, and practices of these professionals has the potential to serve as a model in designing other qualitative studies. The results will have implications for (1) the development of standardized training on sex trafficking for health care providers; (2) the identification of areas for improvement in health care services for those who have been sex trafficked, and (3) a forthcoming Canada-wide survey of health care providers’ knowledge, attitudes, and practices related to sex trafficking, in addition to other subsequent investigations.

### Limitations of study

The proposed study focuses on physicians, nurses, and social workers practicing in health care settings in Ontario. This means that this study will not benefit from expertise and experiences of other professionals working in health care settings and in other provinces of Canada. The results of the study may not be generalizable beyond the cultural context, time, and place in which the interviews will be conducted. While we intend to recruit health care providers with varied professional and social identities, it is possible that we may not obtain a diverse sample. Nonetheless, this study will contribute substantially to the limited body of knowledge of sex trafficking in Canada [7] and augment what is known about the issue globally.

## Supporting information

S1 Checklist Research checklist (COREQ). (PDF)
