# Supplementary material for: A protocol for a qualitative study on sex trafficking: Exploring knowledge, attitudes, and practices of physicians, nurses, and social workers in Ontario, Canada
Source: PLoS One. 2022 Sep 27;17(9):e0274991. doi: 10.1371/journal.pone.0274991 (PMC9514615; doi:10.1371/journal.pone.0274991)
Supplement: S1 Checklist — (PDF) [file pone.0274991.s001.pdf]

*We have taken the below table from: Tong A, Sainsbury P, Craig J. Consolidated criteria for reporting qualitative research (COREQ): a 32-item checklist for interviews and focus groups. Int J Qual Health Care 2007;19(6):349-57.

We have placed an “x” beside the items to which we have reflected on (along with a page number) and have placed a “” beside the items to which we will reflect on continually during data collection, analysis, and writing.

Table 1 Consolidated criteria for reporting qualitative studies (COREQ): 32-item checklist

	No	Item	Guide questions/description
		**Domain 1: Research team and reflexivity**	
		Personal Characteristics	
*	1.	Interviewer/facilitator	Which author/s conducted the interview or focus group?
p. 8 x	2.	Credentials	What were the researcher’s credentials? *E.g. PhD, MD*
p. 8 x	3.	Occupation	What was their occupation at the time of the study?
*	4.	Gender	Was the researcher male or female?
p. 8 x	5.	Experience and training	What experience or training did the researcher have?
		Relationship with participants	
*	6.	Relationship established	Was a relationship established prior to study commencement?
*	7.	Participant knowledge of the interviewer	What did the participants know about the researcher? e.g. *personal goals, reasons for doing the research*
p. 8 x *	8.	Interviewer characteristics	What characteristics were reported about the interviewer/facilitator? e.g. *Bias, assumptions, reasons and interests in the research topic*
		**Domain 2: study design**	
		Theoretical framework	
p. 5 x	9.	Methodological orientation and Theory	What methodological orientation was stated to underpin the study? *e.g. grounded theory, discourse analysis, ethnography, phenomenology, content analysis*
		Participant selection	
p. 6 x	10.	Sampling	How were participants selected? *e.g. purposive, convenience, consecutive, snowball*
p. 6 x	11.	Method of approach	How were participants approached? e.g. *face-to-face, telephone, mail, email*
p. 6 x	12.	Sample size	How many participants were in the study?
*	13.	Non-participation	How many people refused to participate or dropped out? Reasons?
		Setting	
p. 6 x	14.	Setting of data collection	Where was the data collected? e.g. *home, clinic, workplace*
p. 6 x	15.	Presence of non-participants	Was anyone else present besides the participants and researchers?
p. 6 x *	16.	Description of sample	What are the important characteristics of the sample? *e.g. demographic data, date*
		Data collection	
p. 7 x	17.	Interview guide	Were questions, prompts, guides provided by the authors? Was it pilot tested?
*	18.	Repeat interviews	Were repeat interviews carried out? If yes, how many?
p. 6 x	19.	Audio/visual recording	Did the research use audio or visual recording to collect the data?
*	20.	Field notes	Were field notes made during and/or after the interview or focus group?
p. 6 x	21.	Duration	What was the duration of the interviews or focus group?
p. 6, 8 x	22.	Data saturation	Was data saturation discussed?
*	23.	Transcripts returned	Were transcripts returned to participants for comment and/or correction?
		**Domain 3: analysis and findings**z	
		Data analysis	
*	24.	Number of data coders	How many data coders coded the data?
*	25.	Description of the coding tree	Did authors provide a description of the coding tree?
p. 8 x	26.	Derivation of themes	Were themes identified in advance or derived from the data?
p. 8 x	27.	Software	What software, if applicable, was used to manage the data?
*	28.	Participant checking	Did participants provide feedback on the findings?
		Reporting	
*	29.	Quotations presented	Were participant quotations presented to illustrate the themes / findings? Was each quotation identified? e.g. *participant number*
*	30.	Data and findings consistent	Was there consistency between the data presented and the findings?
*	31.	Clarity of major themes	Were major themes clearly presented in the findings?
*	32.	Clarity of minor themes	Is there a description of diverse cases or discussion of minor themes?
